# Enhancing radial strength and expansion uniformity of iron-based vascular scaffolds: a numerical and experimental investigation on topological optimization

**DOI:** 10.3389/fbioe.2025.1736027

**Published:** 2025-12-19

**Authors:** Jia Qiu, Luyao Tang, Wenchao Fu, Li Qin, Deyuan Zhang, Shuhan Wang, Jian Song

**Affiliations:** 1 School of Biomedical Engineering, Shenzhen Campus of Sun Yat-sen University, Shenzhen, China; 2 National and Local Joint Engineering Laboratory of Interventional Medical Biotechnology and System, Biotyx Medical (Shenzhen) Co., Ltd., Lifetech Scientific (Shenzhen) Co., Ltd., Shenzhen, China; 3 Shenzhen Institute for Drug Control, Shenzhen Testing Center of Medical Devices, Shenzhen, China

**Keywords:** expansion homogeneity, finite element analysis, iron-based bioresorbable scaffolds, optimization, radial strength

## Abstract

**Introduction:**

Thin-walled iron-based bioresorbable scaffolds have garnered significant research interest due to their exceptional mechanical properties and favorable biocompatibility. However, current thin-walled iron-based bioresorbable scaffold designs exhibit non-uniform expansion, leading to coating cracking, malapposition, postoperative in-stent restenosis (ISR), and localized pitting corrosion that compromises mechanical integrity.

**Methods:**

This study proposed a dual-factor optimization strategy prioritizing expansion homogeneity through finite element analysis and experimental validation. We systematically modulated the strut width and thickness, as well as the crown radial width in nitrided iron scaffolds, evaluating their mechanical and expansion performance.

**Results:**

It showed that the optimized (OPT) scaffold maintained comparable radial recoil and foreshortening to the original design while demonstrating significant reductions in maximum principal strain (19.2%) and equivalent plastic strain of expand (19.0%).

**Discussion:**

*In vitro* expansion experiments confirmed substantially improved expansion homogeneity, while its radial strength (260.07±4.68 kPa) exceeded that of magnesium/polymer scaffolds, achieving parity with CoCr alloy stents. Enhanced expansion homogeneity mitigates coating fracture risks while maintaining clinically sufficient support.

## 1 Introduction

Cardiovascular disease (CVD) remains a leading cause of global health burden (Deaton et al., 2011; Magnussen et al., 2023; Vogel et al., 2021), with coronary artery disease (CAD) being the primary cause of mortality worldwide (Malakar et al., 2019). Percutaneous coronary intervention (PCI) with vascular stent implantation has become the primary therapeutic approach for obstructive coronary lesions (Wykrzykowska et al., 2017; Wang Z. et al., 2024). Current research on vascular stents has increasingly centered on the development of bioresorbable scaffolds (BRS) to overcome the limitations inherent in conventional drug-eluting stents (DES). Following the completion of their mechanical support function, BRS undergo gradual degradation and are metabolized by the body, thereby facilitating the restoration of natural vascular physiology and mitigating the potential long-term complications associated with permanent implantable devices (Ang et al., 2017; Wiebe et al., 2014). Among various materials explored for BRS, iron-based BRS demonstrates promising clinical potential due to its excellent mechanical properties and favorable biocompatibility. Notably, nitrided iron scaffolds have undergone extensive *in vitro* and *in vivo* investigations (Lin et al., 2017; Zheng et al., 2022; Shen et al., 2021; Deng et al., 2023), which have been the most intensively studied class of iron-based BRS.

Recent research on iron-based BRS has predominantly focused on surface coating modifications to accelerate *in vivo* degradation (Qi et al., 2018; Zhang et al., 2014). However, some studies have shown that thin-walled iron-based BRS may exhibit opening angular non-uniformity of scaffold cells during expansion (Lin et al., 2017; SHI et al., 2021). This may cause scaffold under-expansion and malapposition, resulting in incomplete lesion coverage, coating cracking, vascular injury, and hemodynamic disturbance (Ng et al., 2022; Cornelissen et al., 2023; Beshchasna et al., 2019). These complications collectively elevate risks of in-stent restenosis (ISR) and stent thrombosis (ST) (Hassan et al., 2010; Ullrich et al., 2021). Conventional vascular scaffold design iterations often prioritize parameters such as radial strength, radial recoil, and flexibility, while overlooking the critical aspect of expansion uniformity (Li et al., 2022a; Li et al., 2022b). Therefore, it is essential to conduct topology optimization specifically aimed at improving expansion uniformity, in order to mitigate the adverse effects associated with non-uniform expansion of iron-based BRS.

Finite element analysis (FEA) is a cost-effective approach for structural development of vascular stents, and several studies have been conducted to optimize the structure of iron-based vascular stents based on FEA. For example, Wang et al. (Wang L. et al., 2024) optimized the cross-section of the stent strut by the response surface method to obtain low-nickel stainless steel stents with better compression resistance. Sriram et al. (Tammareddi et al., 2016) optimized the competing objectives during the expansion of 316L stents to minimize the risk of arterial injury during stent implantation and to maintain stent stability. However, these studies focused on optimizing the mechanical properties of the stents and lacked attention to the homogeneity of stent expansion.

In this study, we proposed a dual-factor synergistic optimization strategy, taking expansion uniformity as the primary objective. Through computational modeling and FEA, we systematically evaluated the coupled effects of scaffold width/thickness and crown geometry on the mechanical and deployment performance of iron-based BRS. Our goal was to design a nitrided iron scaffold with improved expansion uniformity while ensuring mechanical performance remains within clinically acceptable ranges. The proposed topology-optimized structure aims to enhance scaffold apposition and preserve coating integrity, thereby improving long-term clinical outcomes such as reduced risks of ISR and ST, as well as promoting more favorable vascular remodeling and physiological vessel healing.

## 2 Materials and methods

### 2.1 Optimization strategy

**FIGURE 1:**
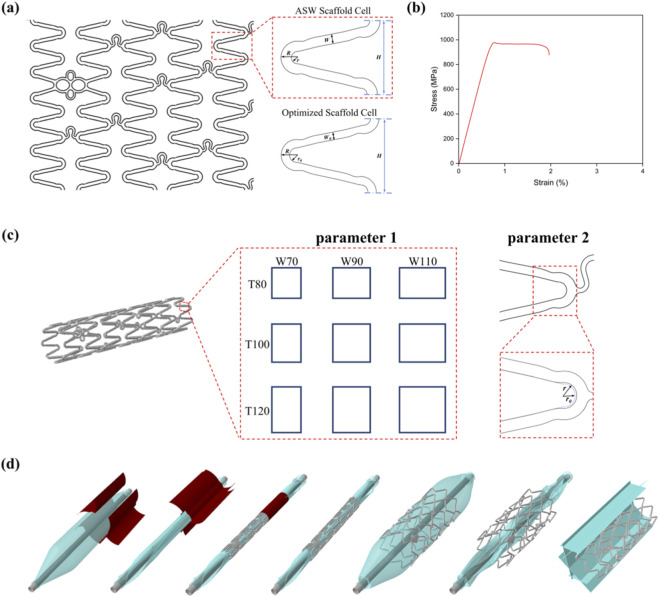
Iron-based BRS topology optimization process and finite element model construction. **(a)** Plan view of the scaffold structure and schematic diagrams of the scaffold unit cells before and after optimization; **(b)** True stress-strain curve of the iron-based scaffold tubing; **(c)** Parameters 1 and 2 in the two-factor structural optimization of the scaffold; **(d)** The entire finite element simulation process of the scaffold, from left to right: three-fold balloon compression, three-fold balloon wing wrapping, scaffold crimping, scaffold recoil after crimping, scaffold expansion, scaffold recoil after expansion, and scaffold radial strength extraction.

The geometry of the scaffold unit is illustrated in Figure 1a. The original iron-based BRS was designed with an asymmetrical sine waveform (ASW) and the U-shaped connectors to obtain better mechanical comprehensive performance. Initially, the effects of varying strut width and wall thickness (parameter 1) on radial strength were assessed based on cross-sectional mechanical performance. The optimal cross-sectional dimensions were determined by taking into account the equivalent plastic strain of expand (PEEQ_expand_), radial recoil (RR) and other deformation metrics. Subsequently, variations in crown geometry (parameter 2) were examined for their impact on plastic deformation and expansion behavior. The final scaffold design was derived by integrating both sets of parameters, and its performance was validated via *in vitro* balloon expansion experiments.

### 2.2 Mechanical properties of scaffold

The nitrided iron tubes were supplied by BiotyxMedical (Shenzhen, Guangdong, China). To characterize the mechanical properties, uniaxial tensile tests were performed on the tubes following the ASTM E8/E8M-11 standard, employing a tensile testing machine (UTM 6104, SUNS Technology). Given that this study involves large plastic deformations, the experimentally obtained engineering stress-strain curve was converted into a true stress-strain curve (Figure 1b) to accurately describe the material’s plastic behavior for simulation (De souza neto et al., 2011).

The nitrided iron scaffold material was then modeled as an isotropic elastoplastic material in the finite element analysis. The key mechanical properties implemented in the model were as follows: Young’s modulus = 21 GPa, yield strength = 836 MPa, and ultimate tensile strength = 976 MPa, with additional parameters provided in Supplementary Table S1.

### 2.3 Finite element simulation

The FEA model included six components: scaffold, balloon, vessel, catheter, rigid compression plates, and crimping surfaces. The balloon was modeled as a linear elastic material with a Young’s modulus of 1,100 MPa and a Poisson’s ratio of 0.4 (Geith et al., 2019). The vessel was modeled as a cylinder with an inner diameter of 2.8 mm and a wall thickness of 0.58 mm. A sixth-order reduced polynomial constitutive model was employed, utilizing the correlation coefficients for the media layer under the assumption of a single-layer arterial wall (Li et al., 2022b; Pant et al., 2012). These coefficients are listed in Supplementary Table S2.

The simulation steps (Figure 1d) included:

1. Crimping the scaffold to 1.086 mm using rigid plates to simulate balloon folding and preserve residual balloon stress.
2. Pressurizing the balloon to 12 atm for scaffold deployment, followed by balloon removal to allow elastic recoil.
3. Gripping the scaffold by eight rigid platens to test the radial strength.

Aiming to balance the computational accuracy and efficiency, a convergence study of the vascular scaffold was conducted (He et al., 2024). The scaffold was meshed with four different element sizes (0.01 mm, 0.015 mm, 0.02 mm, and 0.05 mm) and simulated the whole crimping process by rigid plates. By comparing the slope of the force-diameter curve during the initial linear elastic stage of compression (Supplementary Figure S1), the mesh size was selected as 0.02 mm, as its linear slope difference was 4.4% (i.e., 11.14 N/m for the 0.05 mm mesh size and 11.64 N/m for the 0.06 mm mesh size), which was less than 5%. The scaffold was meshed with 97000–150000 elements due to variations in its geometric parameters across different simulations. The interaction between the scaffold and the balloon, the scaffold and the rigid plates, and the scaffold and the blood vessel was simulated by the general contact algorithm in explicit calculation. The hard contact and the tangential behavior (friction coefficient of 0.2) were set. Finite element analysis was performed using ABAQUS/Explicit 6.14 (Dassault Systèmes, Vélizy-Villacillay, France).

### 2.4 *In Vitro* mechanical testing

Nitrided iron tubes provided by BiotyxMedical were laser-cut into asymmetric sinusoidal waveform scaffolds. Post-processing included chemical polishing and ultrasonic cleaning. The scaffolds were then crimped onto tri-folded balloons with an outer diameter (OD) of 1.0 mm using the scaffold crimping machine (REE31, Blockwise Engineering LLC, United States).

Controlled balloon inflation (0–12 atm) was performed using precision pressure pumps, with scaffold morphological changes recorded via ultra-depth three-dimensional microscope (VHX-970F, KEYENCE, Japan). The radial recoil, longitudinal foreshortening, and metal coverage rate were calculated by using the Equations 1–3:

$$Radial\;recoil=\frac{D_{inflating}-D_{deflated}}{D_{inflating}}\times 100\%$$ (1)

$$Foreshortening=\frac{L_{0}-L_{inflating}}{L_{0}}\times 100\%$$ (2)

$$Metal\;coverage\;rate=\frac{S_{scaffold}}{S_{0}}\times 100\%$$ (3)

Where $D_{inflating}$ and $D_{deflated}$ are the radial diameters of the scaffold at nominal inflating pressure and after balloon unloading, respectively; $L_{0}$ and $L_{inflating}$ are the longitudinal lengths of the scaffold in the crimped state and at nominal inflating pressure, respectively. The $S_{scaffold}$ is the external surface area of the scaffold, $S_{0}$ is the surface area of a cylinder with the same outer diameter and length as the scaffold.

Radial compression testing of the deployed scaffolds was conducted using the MSI RX550 machine according to ASTM F3067-14, with scaffolds compressed to 90% of their outer diameter to determine radial strength.

### 2.5 Statistical analysis

All experimental datasets were statistically presented as mean values ± standard deviation (SD). Each test had at least three independent samples. Statistical analyses were conducted using SPSS Statistics 25 software (IBM, United States) through Student’s t-test. Statistical significance was defined as p < 0.05.

## 3 Results and discussion

### 3.1 Strut width/thickness optimization

**FIGURE 2:**
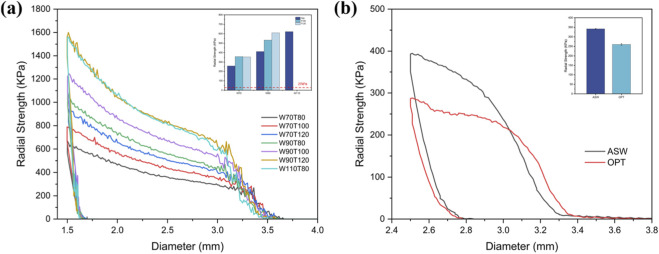
Radial strength of the scaffold. **(a)** Radial strength–outer diameter curve of the scaffold obtained from FEA; the bar chart in the upper right compares radial strength among different scaffold groups in simulation (radial strength exceeding 27 kPa is generally considered sufficient to support the artery); **(b)** Radial strength–outer diameter curve of the scaffold obtained from *in vitro* experiments; the bar chart in the upper right shows the comparison of scaffold radial strength before and after optimization based on *in vitro* experiments.

Figure 2 presents the radial strength of scaffolds with varying strut widths and wall thicknesses. At constant wall thickness, radial strength increased with strut width. Among scaffolds with 110 μm struts, only the W110T80 sample achieved complete expansion under target pressure. Thus, the other two groups were excluded from further analysis. For the current optimization primary objective, PEEQ_expand_ and radial strengths of different groups were compared to ensure radial support while maintaining low stress concentrations to avoid cracking of the iron-based scaffold coatings or their localized corrosion. Given that a radial strength exceeding 27 kPa is generally considered adequate for arterial support (Shen et al., 2021), the radial strength derived from finite element simulations for all samples significantly surpassed 27 kPa. As a result, emphasis was placed on comparing the PEEQ_expand_ results, with the corresponding data matrix displayed in Table 1. The PEEQ_expand_ decreased with increasing scaffold strut thickness and decreasing width. Conversely, the 70 μm width scaffold group exhibited the smallest PEEQ_expand_, which diminished ∼20% compared to the original scaffold sample (W90T100). Based on the previous study (Zahedmanesh and Lally, 2009), the rate of ISR caused by thin-walled scaffolds is lower than that of thick-walled scaffolds. Therefore, the W70T100 configuration was chosen for further optimization.

**TABLE 1:** The FEA resulting matrix of scaffold strut width/thickness optimization.

Sample	Optimization parameters		Target value extraction			
	W (μm)	T (μm)	Radial strength (kPa)	PEEQ_expand_	RR (%)	Coverage (%)
W70T80	70	80	257	0.34	4.55	17.95
W70T100	70	100	357	0.33	4.34	17.95
W70T120	70	120	354	0.33	2.73	17.95
W90T80	90	80	411	0.45	3.03	23.10
W90T100	90	100	532	0.42	4.00	23.10
W90T120	90	120	609	0.4	3.00	23.10
W110T80	110	80	622	0.54	3.12	28.20

*In vitro* tests showed that the optimized (OPT) scaffold, i.e., W70T100, had a 23.92% lower radial strength compared to the ASW scaffold (260.07 ± 4.68 vs. 341.82 ± 2.22 kPa). Despite this, the OPT scaffold demonstrated significantly higher radial strength than contemporary magnesium alloy scaffolds (93–129 kPa) (Li et al., 2022a; Chen et al., 2019) and polylactic acid scaffolds (30–40 kPa) (Agrawal et al., 1992; Wang et al., 2016), which was comparable to that of the CoCr alloy scaffolds (Kumar and Bhatnagar, 2021), demonstrating its superior radial support performance.

### 3.2 Crown radial width optimization

Based on the optimized strut width/thickness values obtained in the previous section, five experimental models were designed by varying the radius r_0_ of the inner concentric arc at the scaffold crown region. The corresponding simulation results were summarized in Table 2, where the parameter Δr denotes the crown radial width of the vascular scaffold. Previous studies have shown that the crown width is the critical parameter affecting the equivalent plastic strain and radial recoil of scaffolds (Hsiao et al., 2012; Kapoor et al., 2024). Therefore, PEEQ_expand_ and RR were extracted from the results for comparison. It was found that when the crown radial width exceeds 70 μm, variations in PEEQ_expand_ become statistically insignificant. Furthermore, RR exhibits a decreasing trend with increasing crown width, indicating enhanced post-expansion structural stability of the scaffold. Based on comparative analysis of PEEQ_expand_ and RR, the R80 sample was selected as the optimal topology-optimized configuration due to its minimal RR while maintaining comparable deformation homogeneity, thereby achieving superior expansion performance.

**TABLE 2:** The FEA resulting matrix of scaffold crown dimensional optimization.

Sample	Optimization parameters			Target value extraction		
	R (μm)	r_0_ (μm)	Δr (μm)	PEEQ_expand_	RR (%)	Coverage (%)
R60	150	90	60	0.29	5.43	17.12
R65	150	85	65	0.28	4.65	17.56
R70	150	80	70	0.33	4.34	17.95
R75	150	75	75	0.36	4.60	18.31
R80	150	70	80	0.34	4.20	18.61

**FIGURE 3:**
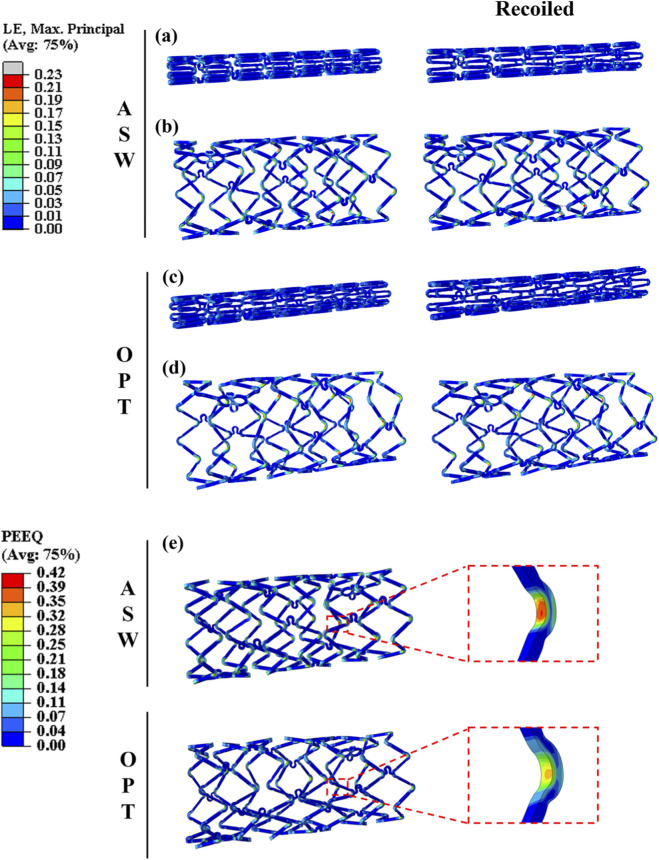
Comparison of strain during ASW and OPT scaffold deployment. Distribution of maximum principal stress for the ASW scaffold **(a,b)** and the OPT scaffold **(c,d)** after crimping (left) and recoil (right); after balloon expansion (left) and subsequent recoil (right). **(e)** Distribution of PEEQ_expand_ for the ASW and OPT scaffolds.

Figures 3a–d presents the simulated results of maximum principal strain (MPS) for the ASW and OPT scaffolds. For the ASW scaffold, the MPS after crimping and recoil reached 0.150 (Figure 3a), whereas the OPT scaffold exhibited a reduced value of 0.132 under the same conditions (Figure 3c). After full expansion and subsequent recoil, the MPS in the ASW scaffold increased to 0.291 (Figure 3b). In contrast, the OPT scaffold showed a lower MPS of 0.235 (Figure 3d), representing a reduction of 19.2%. In addition to MPS, PEEQ is also an important indicator for measuring the uniformity of scaffold expansion and structural durability (Chen et al., 2019). Figure 3e shows the distribution of PEEQ_expand_ before and after scaffold optimization. As indicated by the contour plots, the maximum PEEQ_expand_ values were concentrated at the crown regions of the scaffolds. The ASW scaffold exhibited a peak PEEQ_expand_ of 0.42, while the OPT scaffold demonstrated a lower maximum of 0.34, corresponding to a 19.0% reduction.

Excessive MPS during deployment has been reported to cause endothelial cell detachment due to overstretching beyond physiological limits (Cornelissen et al., 2019), which can expose the underlying basement membrane. This exposure facilitates platelet adhesion and triggers smooth muscle cell migration and proliferation, ultimately leading to neointimal hyperplasia and restenosis (Timmins et al., 2011; Liu et al., 2023). In addition, higher PEEQ/MPS values also increase the risk of fracture during scaffold deployment. Zhang et al. (Zhang et al., 2018) designed a magnesium alloy scaffold based on the structure of a biodegradable polymer scaffold and found residual high stress at the scaffold crown and connection points, which can lead to scaffold fracture. In response, Everett et al. (Everett et al., 2016) used a computational model to evaluate the impact of scaffold design on maximum/minimum principal strain and PEEQ in order to assess scaffold fatigue risk. In summary, the OPT scaffold design in this study reduced MPS/PEEQ, which not only mitigates localized vascular wall injury but may also decrease the subsequent incidence of neointimal hyperplasia and fracture-related complications.

### 3.3 Enhanced expansion homogeneity and superior radial support

**FIGURE 4:**
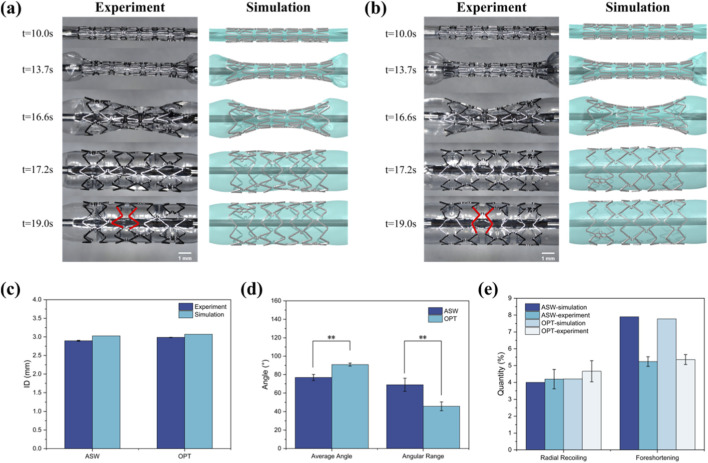
Scaffold expansion morphology and expansion performance. **(a)** Comparison of expansion morphology of the ASW scaffold between *in vitro* experiments (left) and finite element simulations (right); **(b)** Comparison of expansion morphology of the OPT scaffold between *in vitro* experiments (left) and simulations (right); **(c)** Comparison of inner diameters obtained from *in vitro* expansion and finite element simulation; **(d)** Comparison of average opening angles and angular range from *in vitro* scaffold expansion; **(e)** Comparison of radial recoiling and foreshortening values for the ASW and OPT scaffolds obtained from both *in vitro* experiments and simulations.

The expansion morphologies of the ASW and OPT scaffolds during finite element simulation and *in vitro* experimental expansion are shown in Figures 4a,b. The scaffold-balloon deformation results extracted from FEA demonstrated high concordance with experimental observations. For the ASW scaffold, significant variation in strut opening angles was observed after expansion (Figures 4a,d), with an average opening angle of 76.89° ± 3.29° and an angular range of 68.98° ± 7.08°. In contrast, the OPT scaffold exhibited a markedly improved expansion profile, with a mean opening angle of 90.86° ± 1.67° and an angular range reduced to 45.78° ± 4.75°, the angular range representing a 33.63% reduction compared to the ASW scaffold. The increase in average opening angle may be partially attributed to the slight decrease in radial strength. Moreover, the standard deviation of the inner diameter for the OPT scaffold was 0.004 mm (Figure 4c), substantially lower than that of the ASW scaffold (0.013 mm), indicating significantly improved expansion uniformity. These results collectively demonstrate that topology optimization effectively enhances the expansion homogeneity of nitrided iron-based scaffolds. From a mechanical perspective, this optimization enhances scaffold apposition. This can reduce the risk of ISR due to underexpansion (Fujii et al., 2004), as well as the risks of endothelial injury and neointimal hyperplasia caused by overexpansion (Timmins et al., 2011; Liu et al., 2023), thereby comprehensively reducing the risk of postoperative restenosis. Furthermore, most contemporary scaffolds incorporate surface coatings. Improved expansion homogeneity also contributes to preserving coating integrity, preventing localized coating damage due to mechanical stress that would otherwise influence post-implantation corrosion.

**FIGURE 5:**
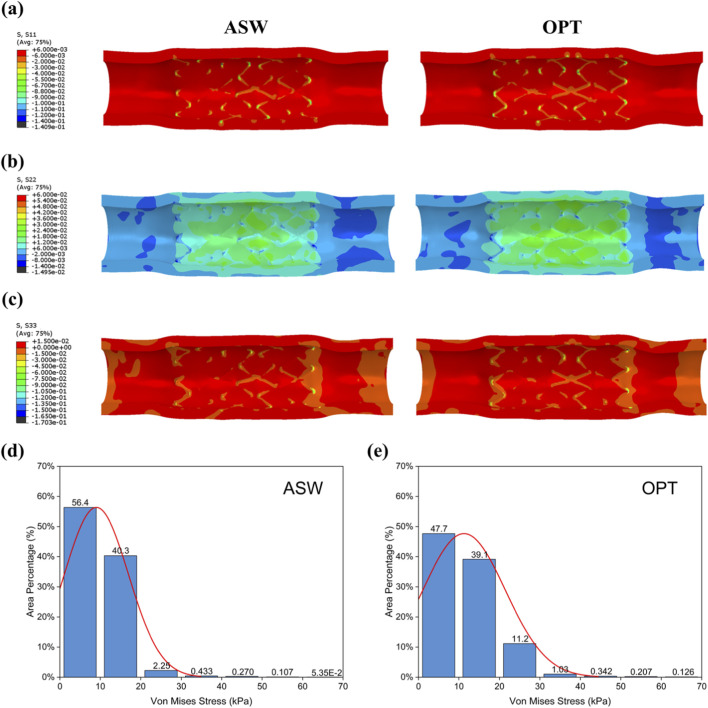
Vascular wall stress results after scaffold implantation. Stress distribution on the vessel wall after scaffold expansion and recoil: **(a)** radial stress; **(b)** circumferential stress; **(c)** axial stress. Area-based histogram of von Mises stress on the luminal surface of the vessel within the implantation region for **(d)** ASW and **(e)** OPT scaffolds.

Furthermore, the optimized scaffold expansion model was deployed into a simplified vascular model and expanded under nominal pressure. The three principal stresses along the cylindrical coordinate directions of the vessel wall were extracted and compared (Figures 5a–c). The results indicate that the optimized scaffold exerted more uniform radial support and provided stronger mechanical support to the vessel wall. Histogram analysis of endothelial surface von Mises stress distributions extracted from Figure 5d reveals that both ASW and OPT scaffolds predominantly induced relatively low intimal stresses (<40 kPa) (Chen et al., 2019). Within the 0–20 kPa range, the area fraction was lower for the OPT scaffold (86.8%) than for the ASW scaffold (96.7%). Conversely, in the 20–30 kPa interval, OPT demonstrated a significantly higher stress area fraction than ASW. Critically, these stress magnitudes remained below documented thresholds for vascular injury while providing adequate mechanical support (Conway et al., 2021). At relatively high stress regions (≥40 kPa), both scaffolds exhibited minimal area fractions (ASW: 0.617%; OPT: 0.97%) with no statistically significant difference.

### 3.4 Relationship between design parameters and performance outcomes

**FIGURE 6:**
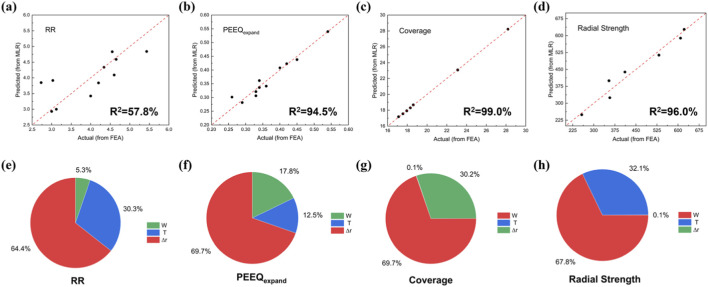
Relationship between design parameters and performance outcomes. Differences between MLR predicted values and FEA results: **(a)** radial recoil (RR); **(b)** equivalent plastic strain of expansion (PEEQ_expand_); **(c)** metal coverage rate; **(d)** radial strength, R^2^ in the lower right corner of the images represents the prediction error metrics for multiple linear regression model. Influence of input factors W, T, and Δr on **(e)** RR, **(f)** PEEQ_expand_, **(g)** coverage, and **(h)** radial strength.

A multiple linear regression (MLR) model was employed to compare differences between predicted results and FEM-derived values. As evidenced by the error plots in Figures 6a–d, all outcomes except RR demonstrated good fitting performance, with R^2^ values exceeding 90% (Figures 6b–d), indicating the model’s effective predictive capability for these responses.

Furthermore, the influence of design parameters on the optimization objectives was assessed, including RR, PEEQ_expand_, radial strength, and metal coverage rate. The results showed that crown radial width constituted the most significant contributor for both RR and PEEQ_expand_ (RR: 64.4%; PEEQ_expand_: 69.7%). For RR, crown radial width represented the primary influencing factor, followed by scaffold thickness (T: 30.3%). Regarding PEEQ_expand_, beyond crown radial width, strut width (W: 17.8%) and thickness (T: 12.5%) exhibited comparable influence magnitudes. Metal coverage rate is a critical factor influencing the endothelialization process of the scaffold, while radial strength affects its post-implantation support performance and expansion behavior. For these two response outcomes, strut width emerged as the primary contributor (Coverage: 69.7%; Radial Strength: 67.8%), consistent with expectations and relevant literature report (Li et al., 2022b).

### 3.5 Limitations and future work

It should be noted that there were some limitations in the presented study. First, the finite element model did not fully account for manufacturing deviations (e.g., geometric deviations and residual stresses in the scaffold) and the nonlinear mechanical behavior of the balloon material, resulting in a discrepancy between the simulated and experimental radial strength values. In addition, constrained by the current methodology, the constructed MLR model was based on a limited dataset, which carries a risk of overfitting. Furthermore, since this study primarily focused on the mechanical behavior and geometric morphology of the scaffold during expansion, the proposed beneficial effect of expansion uniformity on coating integrity and malapposition remains a hypothesis derived from mechanical findings. Future research will focus on developing more accurate finite element models, expanding the dataset to enhance statistical reliability, and validating the clinical translational potential through biological evaluations and *in vivo* studies.

## 4 Conclusion

This study developed a topology-optimized iron-based BRS through finite element simulation. Experimental validation quantified performance differentials between pre- and post-optimization scaffolds, while MLR analysis was employed to investigate the relationships between the design parameters and response outcomes. Based on the results, the key conclusions are as follows.

1. Enhanced scaffold expansion homogeneity can effectively reduce postoperative complications and coating fracture risks. Compared with the ASW scaffold, the OPT scaffold showed a 33.63% reduction in expansion angular range and a 49.24% decrease in the standard deviation of the mean opening angle. Additionally, the OPT scaffold provides more significant yet safe mechanical support within the 20–30 kPa stress range.
2. By increasing the crown radial width and decreasing the strut width, the OPT scaffold achieves a significant reduction in both MPS and PEEQ_expand_ compared to the ASW scaffold. This correlation is supported by the MLR analysis, which identifies the increased crown radial width as the primary factor negatively correlated with PEEQ_expand_.
3. *In vitro* expansion experiments showed that compared with the ASW scaffold, the OPT scaffold exhibited similar RR, FS, and reduced metal coverage rate. Additionally, although the radial strength of the OPT scaffold decreased, it was comparable to CoCr alloy stents.

## Data Availability

The original contributions presented in the study are included in the article/Supplementary Material, further inquiries can be directed to the corresponding authors.
