# Supplementary material for: Enhancing radial strength and expansion uniformity of iron-based vascular scaffolds: a numerical and experimental investigation on topological optimization
Source: Front Bioeng Biotechnol. 2025 Dec 19;13:1736027. doi: 10.3389/fbioe.2025.1736027 (PMC12757364; doi:10.3389/fbioe.2025.1736027)
Supplement: Supplementary file 1 [file Supplementaryfile1.docx]

*Supplementary Information*

**Enhancing Radial Strength and Expansion Uniformity of Iron-Based Vascular Scaffolds: A Numerical and Experimental Investigation on Topological Optimization**

Jia Qiu ^a^, Luyao Tang ^a, b^, Wenchao Fu ^b^, Li Qin ^b^, Deyuan Zhang ^b^, Shuhan Wang ^c*^, Jian Song ^a*^

1. *School of Biomedical Engineering, Shenzhen Campus of Sun Yat-sen University, Shenzhen 518107, China.*
2. *National and Local Joint Engineering Laboratory of Interventional Medical Biotechnology and System, Biotyx Medical (Shenzhen) Co., Ltd., Lifetech Scientific (Shenzhen) Co., Ltd., Shenzhen 518110, China.*
3. *Shenzhen Institute for Drug Control, Shenzhen Testing Center of Medical Devices, Shenzhen 518057, China.*

* Corresponding author: [songj67@mail.sysu.edu.cn](mailto:songj67@mail.sysu.edu.cn) (JS), [wangshuhan@szidc.org.cn](mailto:wangshuhan@szidc.org.cn) (S. Wang)

**Table S1.** Material properties of the model

| **Component** | **Density**  **(ton/mm^3^)** | **Young’s modulus (MPa)** | **Poisson’s ratio** | **Element Type** |
| --- | --- | --- | --- | --- |
| Scaffold | 7.84E-09 | 210000 | 0.34 | C3D8R |
| Balloon^[1]^ | 1.01E-09 | 1100 | 0.4 | C3D8R |
| Vessel^[2]^ | 1.12E-09 | / | / | C3D8R |
| Rigid plate | Rigid body | | | S4R |
| Catheter | Rigid body | | | S4R |
| Crimping surfaces | Rigid body | | | S4R |

**Table S2.** The reduced polynomial coefficients for the vessel model^[3]^

|  | **C10** | **C20** | **C30** | **C40** | **C50** | **C60** |
| --- | --- | --- | --- | --- | --- | --- |
| Vessel | 6.52E-03 | 4.89E-02 | 9.26E-03 | 0.76 | -0.43 | 8.69E-02 |


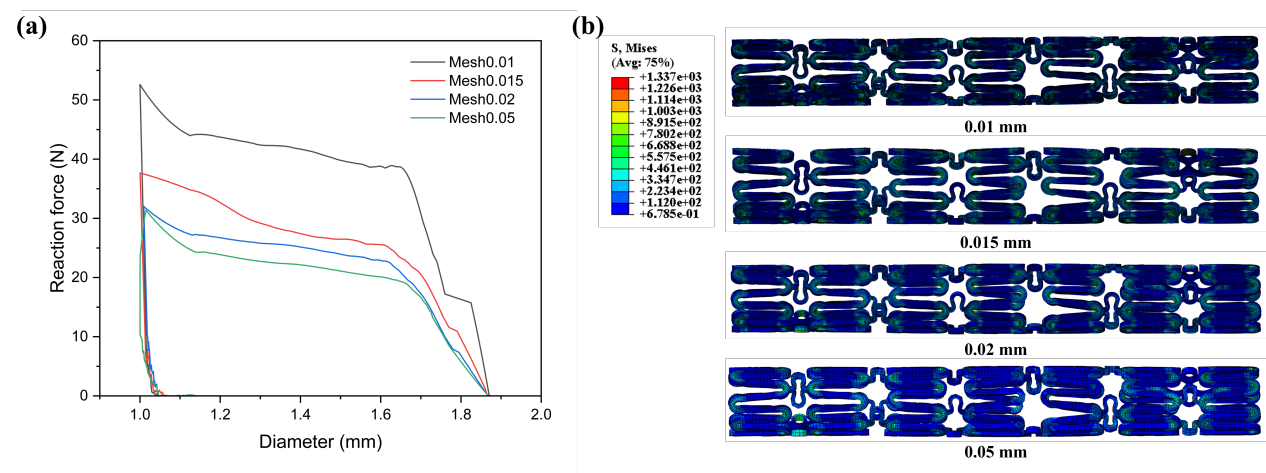


**Fig. S1. Mesh convergence analysis of the iron-based scaffold with four element sizes.** (a) The diameter-force curves of the iron-based scaffold during the whole crimping process, and (b) the stress distribution of scaffold.

[1] GEITH M A, SWIDERGAL K, HOCHHOLDINGER B, et al. On the importance of modeling balloon folding, pleating, and stent crimping: An FE study comparing experimental inflation tests [J]. International journal for numerical methods in biomedical engineering, 2019, 35(11): e3249.

[2] LI Y, WANG J, SHENG K, et al. Optimizing structural design on biodegradable magnesium alloy vascular stent for reducing strut thickness and raising radial strength [J]. Materials & Design, 2022, 220: 110843.

[3] GERVASO F, CAPELLI C, PETRINI L, et al. On the effects of different strategies in modelling balloon-expandable stenting by means of finite element method [J]. Journal of Biomechanics, 2008, 41(6): 1206-12.
